# Determination of Propranolol Hydrochloride in Pharmaceutical Preparations Using Near Infrared Spectrometry with Fiber Optic Probe and Multivariate Calibration Methods

**DOI:** 10.1155/2015/795102

**Published:** 2015-03-12

**Authors:** Jucelino Medeiros Marques Junior, Aline Lima Hermes Muller, Edson Luiz Foletto, Adilson Ben da Costa, Cezar Augusto Bizzi, Edson Irineu Muller

**Affiliations:** ^1^Departamento de Química, Universidade Federal de Santa Maria, 97105-900 Santa Maria, RS, Brazil; ^2^Departamento de Engenharia Química, Universidade Federal de Santa Maria, 97105-900 Santa Maria, RS, Brazil; ^3^Departamento de Biologia e Farmácia, Laboratório de Limnologia, Universidade de Santa Cruz do Sul, 96815-900 Santa Cruz do Sul, RS, Brazil

## Abstract

A method for determination of propranolol hydrochloride in pharmaceutical preparation using near infrared spectrometry with fiber optic probe (FTNIR/PROBE) and combined with chemometric methods was developed. Calibration models were developed using two variable selection models: interval partial least squares (iPLS) and synergy interval partial least squares (siPLS). The treatments based on the mean centered data and multiplicative scatter correction (MSC) were selected for models construction. A root mean square error of prediction (RMSEP) of 8.2 mg g^−1^ was achieved using siPLS (s2i20PLS) algorithm with spectra divided into 20 intervals and combination of 2 intervals (8501 to 8801 and 5201 to 5501 cm^−1^). Results obtained by the proposed method were compared with those using the pharmacopoeia reference method and significant difference was not observed. Therefore, proposed method allowed a fast, precise, and accurate determination of propranolol hydrochloride in pharmaceutical preparations. Furthermore, it is possible to carry out on-line analysis of this active principle in pharmaceutical formulations with use of fiber optic probe.

## 1. Introduction

Propranolol (1-isopropylamino-3-(1-naphthyloxy)-2-propranolol) is a *β*-adrenergic blocking drug belonging to class II of antiarrhythmics that has wide application for the treatment of cardiac arrhythmia, sinus tachycardia, angina pectoris, and hypertension [1]. Due to its therapeutic and pharmacological relevance, propranolol hydrochloride has been determined in pharmaceutical preparations by different techniques, such as spectrophotometry [2–4], spectrofluorimetry [5], electrochemical [6], and liquid chromatography [7, 8]. In addition one of the pharmacopoeia reference methods for propranolol hydrochloride assay is carried out using UV spectrometry with suitable dilution of the sample in methanol [9]. These techniques showed some drawback as the use of high amount of reagent, time consumption, and great generation of chemical residues.

In the last years, continuous increase of near and mid infrared spectrometry (NIR and MID) applications as analytical techniques for the quantification of complex mixtures has been observed [10–21]. A NIR spectrometry with fiber optic probe (FTNIR/PROBE) allows a fast analysis of different mixtures. In addition, this technique is promising for using in Process Analytical Technology (PAT) because it allows on-line quantitative monitoring and fast data acquisition [22].

NIR spectrometry combined with multivariate analysis allows improving the quality of results obtained for complex mixtures by overcoming problems related to overlapped signals. Partial least-squares (PLS) regression is the most popular multivariate calibration method for quantitative analysis because it can resolve overlapping peaks bands and broad spectral bands of NIR spectra [23, 24]. Additionally, some applications have proposed the use of methods for spectral region selection with suitable algorithms to improve the performance of PLS regression [25–33]. In practice, these methods are based on the identification of a spectrum interval that will produce the lowest prediction error. One of these algorithms is the interval partial least squares (iPLS) consisting in the split of the spectrum in smaller intervals. Subsequent, models are constructed for each of these intervals and the root mean square error of cross validation (RMSECV) is calculated for each model. Finally, a comparison between RMSECV value of each model obtained using iPLS and RMSECV of PLS using full spectrum is carried out [29]. On the other hand, in synergy interval partial least squares (siPLS) algorithm PLS regression models using combination of different intervals of the spectrum are carried out [30].

Thus, in this study, the feasibility of FTNIR/PROBE associated with different PLS regression models was evaluated for the propranolol hydrochloride determination in pharmaceutical preparation as an alternative to the recommended method by Brazilian Pharmacopoeia using UV assay. Models using iPLS and siPLS algorithms were evaluated and results obtained using FTNIR/PROBE were compared with those obtained using the recommended procedure described in official pharmacopoeias. Finally some parameters such as precision, linearity, and accuracy were also evaluated.

## 2. Experimental

### 2.1. Samples and Reagents

Ultrapure Milli-Q water (18.2 MΩ cm) and analytical-grade reagents (Merck, Darmstadt, Germany) were used for preparation of standards for UV analysis. Propranolol hydrochloride used in preparation of pharmaceutical preparations was purchased from local pharmacy. On the other hand, propranolol hydrochloride reference material from United States Pharmacopoeia was used in preparation of standards for calibration curve using UV spectrometry.

Thirty-three samples were prepared in laboratory through the mixture of propranolol hydrochloride reference material and excipient mixture (50% of carboximetilcellulose, 48% of starch, 1% of colloidal silicon dioxide, and 1% of magnesium stearate). The concentration of propranolol hydrochloride samples ranged from 57.6 mg g^−1^ to 222.5 mg g^−1^.

Samples were mixed in a cryogenic mill (Spex Certiprep, model 6750 Freezer Mill) using two steps: (i) samples were frozen in liquid argon for 2 min and (ii) mixed/ground for 2 min in cryogenic mill. Samples with particle size less than 80 *μ*m were transferred to 15 mL polypropylene flask and used for the subsequent tests.

### 2.2. UV Spectrometry Reference Method

A spectrophotometer (Shimadzu, Multispec-1501) was used as reference technique for assay of propranolol hydrochloride in pharmaceutical preparation according to Brazilian Pharmacopoeia monograph [9]. Sample mass containing up to 20 mg of propranolol hydrochloride was shaken with 20 mL of water during 10 minutes. Then, 50 mL of methanol was added and shaken for more 10 minutes. Samples were diluted up to 100 mL with methanol, homogenized, and filtered. Samples were suitably diluted and analyzed by UV spectrometry at wavelength of 290 nm. Calibration curve for propranolol hydrochloride was constructed with standard concentration ranging from 0.01 to 0.1 mg mL^−1^. Determination of propranolol hydrochloride was performed in triplicate for pharmaceutical preparations.

### 2.3. Near Infrared Spectrometry with Fiber Optic Probe

**Figure 1:**
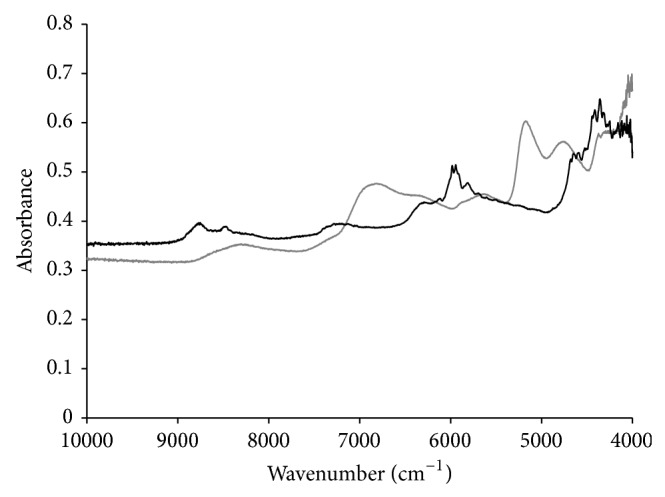
Profile of the spectra of propranolol hydrochloride (black line) and excipients mixture (gray line) using FTNIR/PROBE.

Spectra of sample in powder form were collected in the range from 10000 to 4000 cm^−1^ using a NIR/MID spectrometer (Spectrum 400 FTNIR/MID model, PerkinElmer) with calcium fluoride beam splitter. This instrument was equipped with a NIR fiber optic probe with 2-meter length (FlexIR NIR Fiber Optic Accessory, PIKE Technologies) operated in standard diffuse reflectance sampling tip with inert sapphire window for solid samples. In addition, the NIR fiber optic probe was equipped with low-noise indium-gallium-arsenide detector. The probe was placed inside the polypropylene flask containing the mixtures of propranolol hydrochloride and excipients in order to allow the contact between the sample and the infrared beam. Spectra were obtained in triplicate with 32 scans and 4 cm^−1^ resolution. Medium spectra were used for calibration models construction. Typical spectra of excipients mixture and propranolol hydrochloride are shown in Figure 1.

### 2.4. Construction of Calibration Models

MATLAB software 7.0 version (The Math Works, Natick, USA) was used for constructing of PLS multivariate calibration models. PLS iToolbox 2.0 version was used for variables selection and constructing of multivariate models using iPLS and siPLS algorithms.

Initially, PLS models for propranolol hydrochloride determination in pharmaceutical preparations by FTNIR/PROBE were built using treatment of multiplicative scatter correction (MSC) or first derivative with Savitzky-Golay filter (D). Autoscalling (A) or mean centered data (MC) were used as preprocessing tools for the multivariate calibration models.

In order to improve the predictive ability of the models, PLS regression can be carried out using selected spectral regions containing structural information of propranolol hydrochloride molecule. In this work, the selection of variables for the PLS calibration was carried out using iPLS and siPLS. Interval PLS and synergy interval PLS models were built using spectra division in 10, 20, 30, and 40 intervals. The subinterval or the combined subintervals presenting the minor RMSECV values were selected using iToolbox software.

### 2.5. Evaluation of Models

Root mean square error (RMSE) and correlation coefficient (*R*) were used to characterize and compare predictive abilities of the developed calibration models. Root mean square error of cross validation (RMSECV) was used to select the number of latent variables. Mean square error of prediction (RMSEP) was employed to evaluate the prediction ability of different PLS models.

Comparison between the prediction errors of constructed models was carried out using *F* test (95% confidence level). In addition, accuracy was evaluated using paired *t*-test (95% confidence level). Accuracy was also evaluated using the weighted least square (WLS) which takes into account that the comparable errors in the both axes should fit a straight line where the intercept is not significantly different from 1 [34].

ANOVA (95% confidence level) was used in comparison of results obtained in experiments carried out for precision evaluation.

Systematic error (bias) and standard deviation of validation (SDV) were calculated and systematic error was considered not significant for *t* systematic (*t*_sist_) values lower than critical value (*t*_crit_)_*α*_ = 5% and *n* − 1 degrees of freedom.

Calibration models were also evaluated regarding precision, linearity, and accuracy parameters.

## 3. Results and Discussion

### 3.1. Figures of Merit of UV Spectrometry Reference Method

Coefficient of determination better than 0.99 was obtained and relative standard deviation (RSD) lower than 5% was obtained for determination of propranolol hydrochloride in pharmaceutical preparations. About 300 mL of methanol was required for each sample and sample throughput of 5 samples (in triplicate) per hour was achieved.

### 3.2. Calibration and Prediction Sets

Thirty-three samples were prepared by mixtures of propranolol hydrochloride and excipients. These samples were randomly divided into calibration set (24 samples) and prediction set (9 samples). The concentration of calibration and prediction set ranged from 57.6 to 222.5 mg g^−1^ and from 64.2 to 211.7 mg g^−1^ of propranolol hydrochloride, respectively. Calibration set was used to build the models and prediction set was used to evaluate the predictive ability of the each model.

### 3.3. PLS Models for Propranolol Hydrochloride Determination

Initially, PLS models were built with objective to evaluate different treatments and preprocessing. In Table 1 it is possible to observe the RMSECV values of the PLS models obtained using all variables, different treatment, and preprocessing. The RMSECV values obtained for the different PLS models showed that there were no significant differences in these values (*F* test, 95% confidence level). Moreover, the coefficients of correlation of the PLS models were quite similar (Table 1). Although the models have not showed significant differences in the RMSECV values, the model that used MSC and mean centered data (M) was selected for posterior construction of iPLS and siPLS models, because mean centered data is preferred and most common in literature for construction of models containing spectra data.

### 3.4. iPLS Models

**Figure 2:**
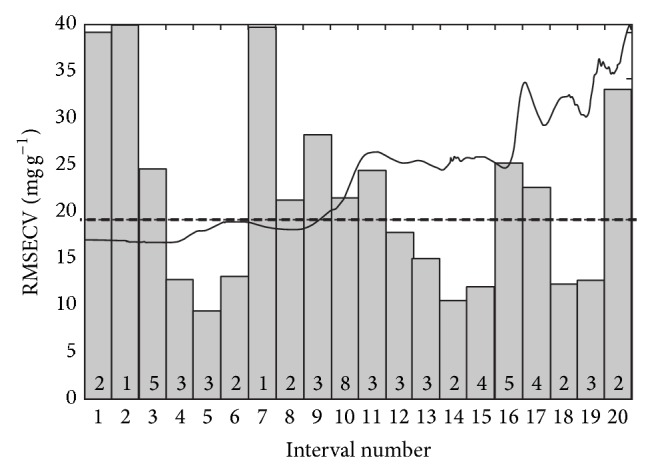
Spectral region selected by interval algorithms for determination propranolol hydrochloride using FTNIR/PROBE. Numbers inside the rectangles were the number of latent variables used in model construction.

Different applications found in the literature have shown that the use of iPLS can improve the predictive ability of the calibration models [26, 27, 29]. The results obtained using iPLS algorithm were presented in a typical figure in order to facilitate the comparison among the RMSECV values obtained for each subinterval model and RMSECV of PLS full model (with all variables) (Figure 2).

In Figure 2 it is possible to observe spectra split in 20 different intervals. The RMSECV value of the PLS full model was plotted in Figure 2 as horizontal black line in order to facilitate the comparison with RMSECV value of each interval. In addition, models built with interval numbers 4, 5, 6, 12, 13, 14, 15, 18, and 19 showed RMSECV values lower than RMSECV value of PLS full model. However, improvement in predictive ability was not observed for iPLS models constructed with spectra split in 10, 20, 30, and 40 intervals because significant differences (*F* test, 95% confidence level) was not observed in RMSEP values obtained for this models.

### 3.5. siPLS Models

In order to decrease the RMSEP value siPLS models were built using combinations of two and three intervals.  Table 2 shows results for propranolol hydrochloride siPLS calibration models. Comparing the different siPLS models obtained it is possible to observe that all siPLS models showed lower RMSECV and RMSEP values when compared with PLS full model. However, significant difference was not observed when RMSEP values of PLS full model and RMSEP values of different siPLS models were compared (*F* test, confidence level 95%).

**Figure 3:**
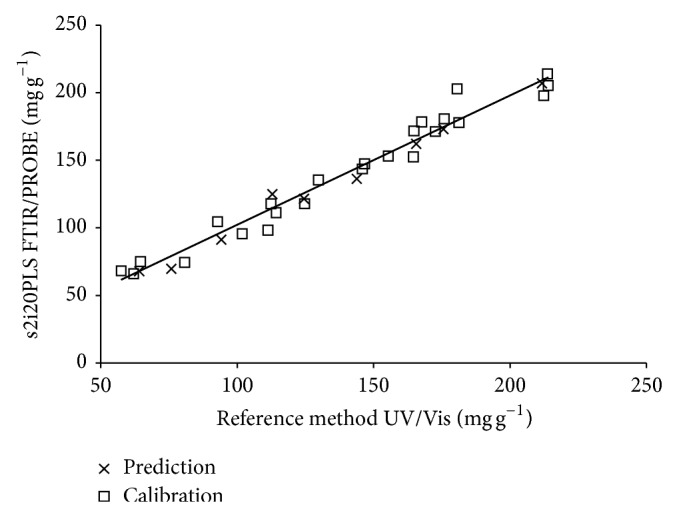
Calibration curve obtained using best model s2i20PLS.

Although no significant differences were observed in RMSEP values for the different siPLS models, model s2i20PLS showed the highest decrease in RMSEP value, that is, about 2 times lower than the RMSEP value of PLS full model. In addition, this model provided correlation coefficient of 0.990 between reference and predicted values (Figure 3) when spectra were divided into 20 intervals and combination of intervals numbers 5 and 16 was used.

The selected intervals included regions of 8501 to 8801 cm^−1^ (interval 5) and of 5201 to 5501 cm^−1^ (interval 16) as can be viewed in Figure 2. Interval 5 corresponds to C–H stretching of second overtone probably related to groups C–H of aromatic ring and CH_3_ that are present in propranolol hydrochloride structure. Interval 16 corresponds to O–H stretching of first overtone and OH stretching + 2x C–O stretching related to structure present in propranolol hydrochloride structure.

Systematic error calculated for the s2i20PLS model was not significant (bias = 3.1 and *t*_sist_ < *t*_crit_). The individual error provided by the s2i20PLS model was smaller than 7% in comparison between propranolol hydrochloride concentration values for proposed method and pharmacopeical methods.

### 3.6. Validation of the Best Calibration Model

The validation based on traditional chemometric parameters such as *R* and RMSEP is insufficient towards pharmaceutical regulatory requirements [35].

The s2i20PLS model was also validated in accordance with the International Conference on Harmonisation (ICH) [36] using parameters usually recommended: precision (repetitively and intermediate precision), linearity, and accuracy.

#### 3.6.1. Precision

The precision of an analytical procedure is usually expressed as the variance, standard deviation, or coefficient of variation of a series of measurements and can be presented as repeatability, intermediate precision, and reproducibility [36]. In this work only repeatability and intermediate precision were evaluated.

Repeatability of the proposed method was performed by the same analyst and applying the method to the same samples six times on the same day. In this work the repeatability was calculated for three different samples with low, medium, and high concentrations of propranolol hydrochloride.

Values for repeatability of the proposed method were lower than 3.63% in concentration of 75.9, 112.9, and 211.7 mg g^−1^. The widely accepted RSD value for this type of determination is 5% [13].

Intermediate precision expresses within-laboratories variations like different equipment, different days, and different analysts. In this work, this parameter was determined by having two analysts for three samples of different concentration and on three different days. The first analyst performed the measurement in the morning while the second analyst measured the sample in the afternoon. In this case, relative standard deviation was lower than 5%. Significant differences between results obtained for different analyst in different days were not observed (two-way ANOVA, 95% confidence level). Finally, relative standard deviation was lower than 4.5% for precision evaluated in different days.

#### 3.6.2. Linearity

Correlation between the values using s2i20PLS model and the reference method was evaluated. Ideally the intercept “*a*” and slope “*b*” should be zero and one, respectively, if there was no systematic error in the calibration equation [36]. For propranolol hydrochloride determination using s2i20PLS model the regression equation was *y* = 0.96*x* + 6.36. The confidence interval for the slope [0.87; 1.04] and for the intercept [−5.60; 18.32] included 1 and zero, respectively. Therefore, the proposed method for propranolol hydrochloride allowed a suitable linearity when it is compared to the reference method.

#### 3.6.3. Accuracy

Accuracy was evaluated by comparison of results obtained using the s2i20PLS model with those obtained by UV reference method. Paired *t*-test was performed between s2i20PLS model values and UV reference values. The experimental *t* value (*t* = 0.86; 95% confidence level) was smaller than the critical *t* value (*t* = 2.31; 95% confidence level) and the s2i20PLS model using FTNIR/PROBE data may be considered accurate.

Additionally, accuracy was also evaluated using the weighted least square (WLS) to obtain the elliptical joint confidence region (EJCR). The WLS method was used with results obtained for s2i20PLS model (prediction set) to verify the accuracy and to check if the proposed methodology is free of systematic error. The WLS results include the theoretical expected value (1, 0), indicating that the proposed methodology is accurate and free of systematic errors.

## 4. Conclusion

Results obtained demonstrated that FTNIR/PROBE associated with multivariate analysis is a convenient method for propranolol hydrochloride determination in pharmaceutical preparations. Results obtained using siPLS models for determination of propranolol hydrochloride in powder pharmaceutical preparations showed suitable prediction capacity (lower RMSEP). Variable selection methods were able to produce better models in comparison to the PLS full-spectrum model. Results obtained for propranolol hydrochloride using the better siPLS (s2i20PLS) model were in agreement with results obtained by reference method. The proposed procedure using FTNIR/PROBE and PLS algorithms is faster, less expensive, accurate, and precise and minimizes solvent use when compared to pharmacopoeia UV method.

## Figures and Tables

**Table 1:** Results obtained using full-spectrum PLS model for propranolol hydrochloride determination by FTNIR/PROBE.

Model	VN^a^	Interval	LV^b^	*R*	RMSECV (mg g^−1^)
PLS (D/M)	6000	all	8	0.941	18.4
PLS (D/A)	6000	all	9	0.950	17.9
PLS (MSC/M)	6000	all	7	0.951	17.4
PLS (MSC/A)	6000	all	6	0.953	17.6

^a^VN: total variable numbers; ^b^LV: latent variables.

**Table 2:** Results obtained using siPLS algorithm for propranolol hydrochloride determination by FTNIR/PROBE.

Model	VN^a^	Interval	LV^b^	*R*	RMSECV (mg g^−1^)	RMSEP (mg g^−1^)
PLS (MSC/M)	6000	all	5	0.955	18.4	18.6
s2i10PLS	600	3, 8	6	0.978	9.7	8.7
s2i20PLS	300	5, 16	4	0.990	8.8	8.2
s2i30PLS	200	7, 12	5	0.984	8.3	10.8
s2i40PLS	150	9, 32	4	0.984	8.3	11.7
s3i10PLS	600	3, 7, 9	6	0.979	9.6	13.7
s3i20PLS	300	5, 14, 18	7	0.983	8.7	11.5
s3i30PLS	200	7, 22, 27	8	0.983	8.5	12.0
s3i40PLS	150	9, 22, 32	9	0.986	7.9	14.5

^a^VN: total variable numbers; ^b^LV: latent variables.
